# Effect of amoxicillin on the gut microbiome of children with severe acute malnutrition in Madarounfa, Niger: a retrospective metagenomic analysis of a placebo-controlled trial

**DOI:** 10.1016/S2666-5247(23)00213-6

**Published:** 2023-11

**Authors:** Drew J Schwartz, Amy Langdon, Xiaoqing Sun, Céline Langendorf, Fatou Berthé, Rebecca F Grais, Indi Trehan, Sheila Isanaka, Gautam Dantas

**Affiliations:** a The Edison Family Center for Genome Sciences & Systems Biology, Washington University School of Medicine in St Louis, St Louis, MO, USA; b Center for Women's Infectious Disease Research, Washington University School of Medicine in St Louis, St Louis, MO, USA; c Department of Pediatrics, Washington University School of Medicine in St Louis, St Louis, MO, USA; d Department of Molecular Microbiology, Washington University School of Medicine in St Louis, St Louis, MO, USA; e Department of Obstetrics & Gynecology, Washington University School of Medicine in St Louis, St Louis, MO, USA; f Clinical Research Training Center, Washington University School of Medicine in St Louis, St Louis, MO, USA; g Department of Pathology & Immunology, Washington University School of Medicine in St Louis, St Louis, MO, USA; h Department of Biomedical Engineering, Washington University School of Medicine in St Louis, St Louis, MO, USA; i Departments of Pediatrics, Global Health, and Epidemiology, University of Washington, Seattle, WA, USA; j Department of Research, Epicentre, Paris, France; k Epicentre Niger, Niamey, Niger; l Department of Nutrition, Harvard T H Chan School of Public Health, Boston, MA, USA

## Abstract

**Background:**

Children with severe acute malnutrition are treated with antibiotics as outpatients. We aimed to determine the effect of 7 days of amoxicillin on acute and long-term changes to the gut microbiome and antibiotic resistome in children treated for severe acute malnutrition.

**Methods:**

We conducted a secondary analysis of a randomised, double-blinded, placebo-controlled trial (NCT01613547) of amoxicillin in children (aged 6–59 months) with severe acute malnutrition treated as outpatients in Madarounfa, Niger. We randomly selected 161 children from the overall cohort (n=2399) for initial 12-week follow-up from Sept 23, 2013 to Feb 3, 2014. We selected a convenience sample of those 161 children, on the basis of anthropometric measures, for follow-up 2 years later (Sept 28 to Oct 27, 2015). Children provided faecal samples at baseline, week 1, week 4, week 8, week 12, and, for those in the 2-year follow-up cohort, week 104. We conducted metagenomic sequencing followed by microbiome and resistome profiling of faecal samples. 38 children without severe acute malnutrition and six children with severe acute malnutrition matching the baseline ages of the original cohort were used as reference controls.

**Findings:**

In the 12-week follow-up group, amoxicillin led to an immediate decrease in gut microbiome richness from 37·6 species (95% CI 32·6–42·7) and Shannon diversity index (SDI) 2·18 (95% CI 1·97–2·39) at baseline to 27·7 species (95% CI 22·9–32·6) species and SDI 1·55 (95% CI 1·35–1·75) at week 1. Amoxicillin increased gut antibiotic resistance gene abundance to 6044 reads per kilobase million (95% CI 4704–7384) at week 1, up from 4800 (3391–6208) at baseline, which returned to baseline 3 weeks later. 35 children were included in the 2-year follow-up; the amoxicillin-treated children (n=22) had increased number of species in the gut microbiome compared with placebo-treated children (n=13; 60·7 [95% CI 54·7–66·6] *vs* 36·9 [29·4–44·3]). Amoxicillin-treated children had increased *Prevotella* spp and decreased *Bifidobacterium* spp relative to age-matched placebo-treated children, indicating a more mature, adult-like microbiome.

**Interpretation:**

Amoxicillin treatment led to acute but not sustained increases in antimicrobial resistance genes and improved gut microbiome maturation 2 years after severe acute malnutrition treatment.

**Funding:**

Bill & Melinda Gates Foundation; Médecins sans Frontières Operational Center Paris; National Institute of Allergy and Infectious Diseases; National Institute of General Medical Sciences; Eunice Kennedy Shriver National Institute of Child Health and Human Development; Edward Mallinckrodt Jr Foundation; Doris Duke Foundation.

## Introduction

Severe acute malnutrition has poor clinical and developmental outcomes including acute increases in infections, death, long-term impairment in linear growth, and diminished economic productivity.^1^ Severe acute malnutrition, also known as severe wasting, affected at least 13·6 million children younger than 5 years in 2020 and increased mortality risk by up to 50% for other leading causes of death, contributing to more than half a million deaths per year.^2^ Accordingly, numerous interventions—such as preventive nutritional supplementation and improved water, sanitation, and hygenic practices—have been sought to decrease the incidence of severe acute malnutrition and improve nutritional recovery and mortality during treatment.^1^ Effective treatment exists;^1^ however, relapse and mortality have been reported after successful discharge.^3^ These data suggest that anthropometric restoration alone might be insufficient for complete recovery.

Antibiotics are standard of care for severe acute malnutrition, empirically given because even if children do not present with an obvious disease, they can suddenly deteriorate with an acute infection.^1^ Short-course (ie, 7-day) amoxicillin administration, depending on the setting, has been shown to have a benefit in reducing all-cause mortality, hospitalisation, diarrhoeal illness and improvement in anthropometric measures relative to placebo in children with severe acute malnutrition in Malawi^4^ and Niger.^5^ However, uncertainty still exists on the potential consequences (eg, development of antibiotic resistance and microbiome disruption) of antibiotic treatment in children with severe acute malnutrition.^6^ One hypothesis for the benefit of antibiotics has been a reduction in subclinical or clinical diarrhoeal or respiratory infections.^4^, ^5^, ^7^ Alternately, it is possible that the beneficial effects of antibiotics in this population act through alteration of the gut microbiome. In healthy children, the gut microbiome steadily increases in taxonomic and functional diversity until aged 3 years, with the most pronounced changes occurring during weaning.^8^, ^9^ By contrast, the gut microbiome of a child who is malnourished is age regressed—ie, the gut microbiome resembles that of a younger child.^10^, ^11^ It is currently unknown what the effect of a 7-day course of amoxicillin is on short-term and long-term microbiome and antibiotic resistance genes (ARGs) abundance after treating children for uncomplicated severe acute malnutrition. This information is crucial to understanding the implications of current recommendations for the continued use of routine antibiotics in the management of uncomplicated severe acute malnutrition.

**Research in context**

**Evidence before this study**

We searched PubMed from database inception to Jan 1, 2023, using the search terms “severe acute malnutrition” and “antibiotics” and “severe acute malnutrition” and “antibiotic” and (“resistome” OR “microbiome”). No language restrictions were placed on the search. We found 245 articles. Antibiotics are standard of care in the outpatient management of severe acute malnutrition. 61 studies examined the effect of different antibiotic regimens or placebo on anthropometric outcomes and only six studies examined the short-term (<12 weeks) effect on the gut microbiome or resistome. Antibiotics select for antibiotic-resistant Enterobacteriaceae, but the persistence of these findings with microbiome maturation are unknown.

**Added value of this study**

Our study adds short-term and long-term data on the effect of an antibiotic course versus placebo on the gut microbiome and resistome. We show that a 7-day course of amoxicillin transiently increased antibiotic resistance genes and decreased taxonomic diversity and richness in the gut microbiome, but these changes resolved 3 weeks after treatment concluded. 2 years after initial treatment, children treated with amoxicillin had increased gut microbiome diversity and richness relative to placebo-treated controls without maintaining the previously observed antibiotic resistance gene increase.

**Implications of all the available evidence**

Our work demonstrates no sustained increase in gut microbiome resistance with a short course of amoxicillin. Conversely, antibiotic use was associated with a more mature microbiome 2 years after treatment, suggesting that antibiotics for outpatient treatment of severe acute malnutrition does not decrease long-term microbiome diversity or increase antimicrobial resistance.

Here, we present a gut microbiome and resistome analysis on longitudinally collected faecal samples from children included in a placebo-controlled trial of amoxicillin for outpatient treatment of severe acute malnutrition in Niger.^5^, ^12^

## Methods

### Study design and participants

We performed a secondary analysis of data collected as part of a previously published randomised, double-blinded, placebo-controlled trial (NCT01613547).^5^, ^12^ The original trial (n=2399) evaluated the effectiveness of amoxicillin versus placebo on nutritional recovery from uncomplicated severe acute malnutrition in Madarounfa, Niger. Assignment to the amoxicillin group was randomly allocated and participants and researchers were masked to assignment groups.

For our microbiome analysis, we selected 161 children from the original trial to provide faecal samples during the initial 12-week follow-up period from Sept 23, 2013 to Feb 3, 2014 (appendix 1). We randomly selected these children by selecting one child per working day per group. Of the 161 children in the microbiome substudy, we revisited a convenience sample of children for long-term follow-up and faecal sampling at 2 years post enrolment as a convenience sample from Sept 28 to Oct 27, 2015. These children were selected from the upper and lower quartiles of mid-upper arm circumference (MUAC) and weight for height (WHZ) improvements at 4 weeks. We chose these individuals instead of a random subset because we wanted to include roughly equal proportions of four possible outcomes from the original trial: amoxicillin or placebo treatment with or without nutritional recovery. Specifically, 49% (17 of 35 children) improved their MUAC more than 1 cm or WHZ more than 2·5, whereas the other 51% (18 of 35) improved their MUAC by 0·5 cm or less or WHZ by 2 or less at 4 weeks of follow-up in the original trial.^5^

To account for possible microbiome drift over the 2-year interval in the overall environment, we additionally recruited Nigerien children with severe acute malnutrition (n=6) and Nigerien children without severe acute malnutrition but who did not meet criteria for severe acute malnutrition as reference controls. We collected a single faecal sample with the same sample collection procedures from these six children with severe acute malnutrition and 38 children without severe acute malnutrition (WHZ greater than or equal to –3 and MUAC greater than 125 mm)^13^ from the same or neighbouring household from Sept 30 to Nov 18, 2015 matched to the original participant's age at enrolment 2 years prior. We chose to evaluate baseline age-matched children because of previous studies demonstrating an adult-like microbiome conformation in healthy children^8^ and to confirm that children with severe acute malnutrition maintained an age-regressed microbiome at 2-year follow-up, as previously reported.^10^, ^11^

The study protocol of the original trial was approved by the Comité Consultatif National d'Éthique (Niger) and Comité de Protection des Personnes (France). Written informed consent was obtained from each child's guardian. The Comité Consultatif National d'Éthique (Niger) approved an amendment specific for the additional microbiome sampling and analysis in August 2015.

### Sample collection and processing

Faecal samples were collected during home visits at the scheduled timepoints: week 0 (baseline), week 1, week 4, week 8, week 12, and, for the 2-year follow-up cohort, week 104. Initial collection was in sterile, plastic 50 mL containers, which were placed in a cooler (2–8°C) for transportation back to the Epicentre Maradi laboratory (Maradi, Niger) within 8 h. At the laboratory, stool samples were aliquoted into prelabelled cryovials and then frozen at –80°C. After conclusion of the original trial in 2014, all collected samples were shipped on dry ice to Washington University School of Medicine (St Louis, MO, USA). The samples collected at 2 years were shipped in January, 2016. 0·25 g stool was thawed once directly into the DNA extraction and sequencing pipeline using the Illumina Hi-Seq platform, as previously described.^14^

### Taxonomy and resistance gene prediction

Shotgun metagenomic sequences were quality profiled with FastQC (version 0.11.9), demultiplexed, trimmed, and filtered using Trimmomatic (version 0.33) with the following parameters: leading and trailing sequences of 10 bp, with a sliding window between 4 bp and 20 bp, and minimum length of 60 bp. The hsref38 database on Deconseq (version 0.4.3) was used to screen out any human DNA. Samples with processed read counts below 200 000 were excluded. After processing and excluding samples below 200 000 reads, faecal samples were sequenced to a mean depth of 5·1 million reads (SD 2·3 million). We used the ZymoBIOMICS Microbial Community Standard (catalogue number D6305) as an extraction, sequencing, and processing control. We did not use a negative control for sequencing. MetaPhLan3 (version 3.0.7) was used to assign taxonomy to the shotgun metagenomes.^15^ For plotting relative abundances, taxa with less than 1% abundance were grouped into the 'Other’ category for display only.

### Outcomes

The primary outcome was to identify short-term (ie, 12 weeks) and long-term (ie, 2 years) changes to the microbial taxonomy and resistance gene burden in the gut microbiome following a 7-day course of amoxicillin or placebo. Secondary outcomes were to model microbial and ARG dynamics, determine the association with WHZ and MUAC scores, and associations between microbes and anthropometric outcomes.

### Statistical analysis

WHZ score was calculated with the anthro package in R (version 1.0.0) or AnthroPlus if aged 60 months or older. WHZ score was not calculated for children from whom age, weight or height data were absent, affecting one amoxicillin-treated and two placebo-treated children each at a single timepoint.

We used principal coordinate analyses of Bray-Curtis dissimilarity matrices to determine β diversity differences between samples using the R package 'vegan’ (version 2.6-2). ShortBRED protein markers were built from the Comprehensive Antibiotic Resistance Database (CARD; version 3.0) database using shortbred-identify.py with cluster identity 95% and screened against Uniref90 with a markerLength of 8–300 amino acids.^16^ ShortBRED (version 0.9.4) with the command ShortBRED-quantify was used to determine ARGs in each sample against a curated database including the CARD and AMRFinderPlus on NCBI combined with functionally validated ARGs identified in previous studies.^14^, ^16^, ^17^ This program normalises reads based on marker length and read depth to quantify abundance in reads per kilobase million (RPKM) in metagenomic data. ARGs were categorised according to mechanism and then by gene family as available in the CARD 3.0; ARG association with bacterial species was determined based on CARD annotation.^18^

Demographic and clinical variables and relative abundance measures were analysed with pairwise Wilcox tests with Benjamini-Hochberg correction for multiple comparisons within each timepoint. Linear mixed effect models on randomised (amoxicillin *vs* placebo) longitudinal analyses and non-randomised (amoxicillin *vs* placebo *vs* SAM and non-SAM reference controls) were used to determine differences in microbiome and resistome richness and diversity and WHZ and MUAC over time. Post-hoc pairwise comparisons of linear mixed effect models were performed with a Tukey Honest significant difference adjustment to control for multiple comparisons to limit false positive associations. To determine significant differences in gut microbiome and resistome while controlling for age, WHZ, and MUAC, we used generalised linear mixed-effect models with the MaAsLin2 R package^19^ run with default parameters including a minimum taxa prevalence of 10%. Fixed effects in our models included the interaction of timepoint and treatment (“Description”), the age of the child at each visit (“visage”), WHZ score (“visWHZ”), MUAC (“visMUAC”), change in WHZ score from baseline (“visdelWHZ”), and change in MUAC from baseline (“visdelMUAC”). We used individual (“idno”) as a random effect to control for sampling bias in our randomised analysis because every child did not provide a sample at each timepoint. We used the randomForest package in R (version 4.71.1) to determine the microbial taxa that associated with age in our non-SAM reference children recruited at the 2-year timepoint. We used the parameters “ntree=10,000” and performed 10-fold cross-validation with a 0·5 step with the “rfcv” command. We plotted model error versus the number of explanatory variables of taxa versus age and chose the minimum of both values for the best fit model as previously described.^10^, ^11^ p<0·05 was considered significant for gut microbiome profiling and Benjamini-Hochberg-corrected p values yielding false discovery q values of q<0·1 were considered significant for MaAsLin2 analysis. Because of the complexity of microbiome associations, we wanted to limit false positives; therefore, we corrected for multiple comparisons between metadata variables and microbiome and resistome features in generalised linear mixed effect models with MaAsLin2.^19^ Principal coordinate analysis differences were determined using a PERMANOVA on Bray–Curtis distance with the adonis2 function with subsequent individual component differences determined by pairwise Wilcoxon and Benjamini-Hochberg correction. Analysis was performed in R (version 4.1.1) and R studio (version 2021.09.0).

Shannon diversity index (SDI) was used to determine the diversity within the gut microbiome. We considered a difference of ten species and 0·6 SDI to represent a significant change in the gut microbiome.^9^, ^17^, ^20^

### Role of the funding source

The funders provided financing, but did not play a role in the collection, analysis, or interpretation of data, nor did they contribute to writing the report or the decision to submit for publication.

## Results

**Figure 1:**
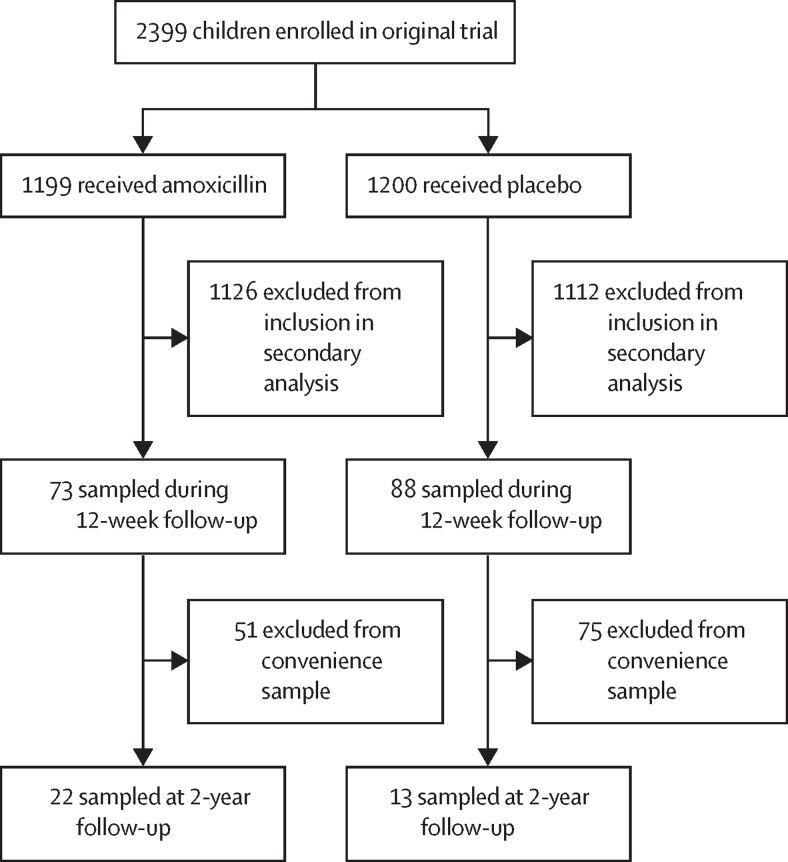
Study profile

We performed shotgun metagenomic sequencing on 353 faecal samples from 161 children during the initial 12-week follow-up period (figure 1). We excluded four samples from metagenomic analyses due to low read counts. We additionally collected a faecal sample from 35 of these children 2 years after baseline (figure 1; appendix 1). Characteristics of the study population are shown in the table.

**Table:** Faecal samples above readcount threshold and clinical metadata collected at each timepoint

		**Baseline**		**Week 1**		**Week 4**		**Week 8**		**Week 12**		**Week 104**			
		Amoxicillin	Placebo	Amoxicillin	Placebo	Amoxicillin	Placebo	Amoxicillin	Placebo	Amoxicillin	Placebo	Amoxicillin	Placebo	Reference control (without severe acute malnutrition)	Reference control (with severe acute malnutrition)
Faecal samples		35	32	39	33	35	43	30	27	41	34	22	13	38	6
Sex															
	Female	19 (54%)	12 (37.5%)	22 (56%)	13 (39%)	21 (60%)	17 (40%)	16 (53%)	12 (44%)	22 (54%)	16 (47%)	12 (55%)	4 (31%)	17 (45%)	3 (50%)
	Male	16 (46%)	20 (62.5%)	17 (44%)	20 (61%)	14 (40%)	26 (60%)	14 (47%)	15 (56%)	19 (46%)	18 (53%)	10 (45%)	9 (69%)	21 (55%)	3 (50%)
Age															
	Median, months	11·0 (8·0 to 21·5)	11·5 (8·0-19·5)	12·3 (8·3 to 21·3)	12·3 (8·3 to 19·3)	12·0 (9·0 to 20·0)	12·0 (9·0 to 21·0)	14·0 (12·0 to 24·0)	13·0 (10·0 to 23·0)	15·0 (12·0 to 25·0)*	13·0 (11·0 to 18·5)*	36·0 (32·0 to 46·0)	38·5 (34·0 to 43·3)	12·0 (9·0 to 22·0)	10·5 (8·5 to 14·0)
	<12	18 (51%)	16 (50%)	19 (49%)	15 (45%)	17 (49%)	20 (47%)	7 (23%)	10 (37%)	8 (20%)	13 (38%)	0 (0%)	0 (0%)	17 (45%)	4 (66%)
	≥12	17 (49%)	16 (50%)	20 (51%)	18 (55%)	18 (51%)	23 (53%)	23 (77%)	17 (63%)	33 (80%)	21 (62%)	22 (100%)	13 (100%)	21 (55%)	2 (33%)
WHZ															
	Median	−3·2 (−3·4 to −2·8)	−3·1 (−3·5 to −2·6)	−2·1 (−2·5 to −1·7)	−2·2 (−3·1 to −1·8)	−1·4 (−1·9 to −0·8)	−1·6 (−2·3 to −1·0)	−1·0 (−1·3 to −0·3)*	−1·4 (−1·6 to −0·6)*	−0·7 (−1·5 to −0·2)	−1·1 (−1·4 to −0·4)	−0·2 (−0·9 to 0·9)	−0·2 (−1·1 to 0·3)	−1·0 (−1·3 to 0·5)	−3·2 (−3·3 to −3·1)
	Change from baseline	NA	NA	1·1 (0·8 to 1·4)*	0·6 (0·2 to 1·1)*	1·5 (1·1 to 2·0)	1·3 (0·8 to 2·0)	2·3 (1·8 to 2·7)*	1·7 (0·9 to 2·5)*	2·3 (1·7 to 3·1)	2·0 (1·4 to 2·6)	3·3 (2·3 to 3·8)	3·1 (1·9 to 3·5)	NA	NA
MUAC															
	Median	11·3 (11·0 to 11·4)	11·2 (10·8 to 11·4)	11·7 (11·4 to 12·0)	11·4 (11·0 to 11·8)	12·0 (11·8 to 12·3)	11·6 (11·2 to 12·4)	12·5 (12·2 to 13·0)†	11·9 (11·7 to 12·1)†	12·5 (12·2 to 13·4)*	12·3 (11·8 to 12·6)*	14·6 (14·0 to 15·4)	14·0 (13·8 to 14·7)	13·9 (13·5 to 14·8)	11·7 (11·5 to 12·1)
	Change from baseline	NA	NA	0·4 (0·3 to 0·6)	0·3 (0·0 to 0·4)	0·7 (0·5 to 1·0)	0·6 (0·2 to 1·1)	1·2 (1·0 to 1·6)*	0·9 (0·6 to 1·4)*	1·3 (0·9 to 1·9)	1·1 (0·7 to 1·5)	3·7 (2·8 to 4·2)	3·5 (2·6 to 4·0)	NA	NA

Data are n, n (%), or median (IQR). All p-values are in appendix 2 (tab 1), statistics determined with pairwise Wilcox test and χ^2^ at each timepoint with shared symbol indicating statistical significance. MUAC=mid-upper arm circumference. WHZ=weight for height z score.

* p<0·05.

† p<0·0001.

Amoxicillin-treated children showed increased improvement over baseline in WHZ score (1·1 *vs* 0·6, p=0·020) and MUAC (0·4 *vs* 0·3, p=0·020) 1 week after baseline compared with placebo-treated children (table; appendix 2 tab 1), consistent with the overall trial (appendix 3 p 2).^5^

**Figure 2:**
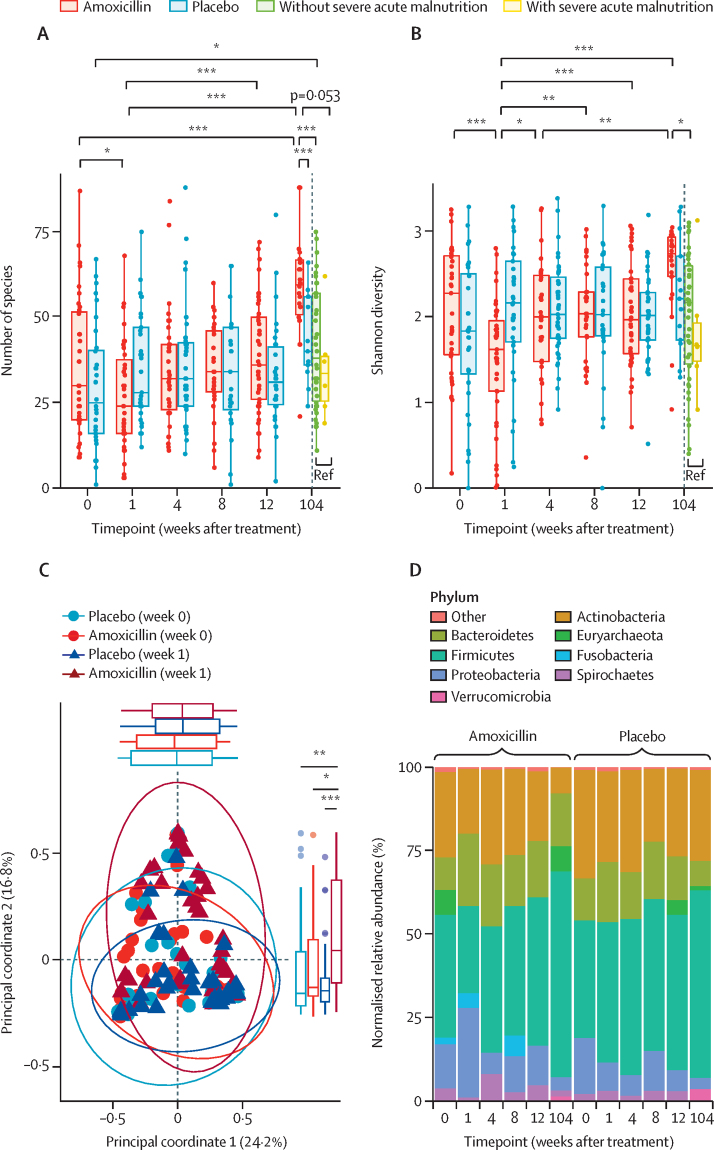
Richness and diversity of the gut microbiome (A) Number of species. (B) Shannon diversity. Solid red line demarcates older placebo-treated and amoxicillin-treated children from younger SAM and non-SAM reference controls. (A–B) p-values determined using linear mixed effect models with subject as the random effect. (C) Bray-Curtis dissimilarity of baseline (week 0) and week 1 samples. The distribution of points in principal coordinate 1 is depicted at the top of the graph, and principal coordinate 2 is depicted to the right with the percent of variance of each principal coordinate in parentheses. Principal coordinate differences determined overall by PERMANOVA and pairwise Wilcoxon test with Benjamini-Hochberg correction for principal coordinate 1 and principal coordinate 2 independently. (D) Taxonomic compositions at the phylum level. Significant values and effect sizes are shown in appendix 2 (tab 2). *p<0·05. **p<0·01. ***p<0·001.

We next wanted to investigate changes in the gut microbiome and resistome accompanying amoxicillin treatment. Amoxicillin-treated children had decreased gut microbiome richness from 37·6 species at baseline to 27·7 at week 1 (figure 2A; estimate 9·9 species decrease [95% CI 4·4–15·4], p=0·024; appendix 2 tab 2). Similarly, SDI decreased from 2·2 at baseline to 1·6 at week 1 (figure 2B; estimate 0·63 [95% CI 0·4–0.9], p=0·0001). We found no overall compositional differences between baseline and week 1 with Bray-Curtis dissimilarity (figure 2C, PERMANOVA p=0·55); however, we observed a significant difference in week 1 amoxicillin gut microbiomes compositionally in principal coordinate 2 from week 0 amoxicillin (p=0·012) and week 1 placebo (p=0·0008; appendix 2 tab 2). Firmicutes was the most abundant phylum for the placebo and amoxicillin groups at baseline (35% *vs* 37%; figure 2D). Proteobacteria increased at the end of amoxicillin therapy to 27% relative abundance, increased from 13% at baseline (p=0·051) and higher than the placebo-treated group at baseline (17%, p=0·051) and at week 1 (9%, p=0·018; figure 2D).

**Figure 3:**
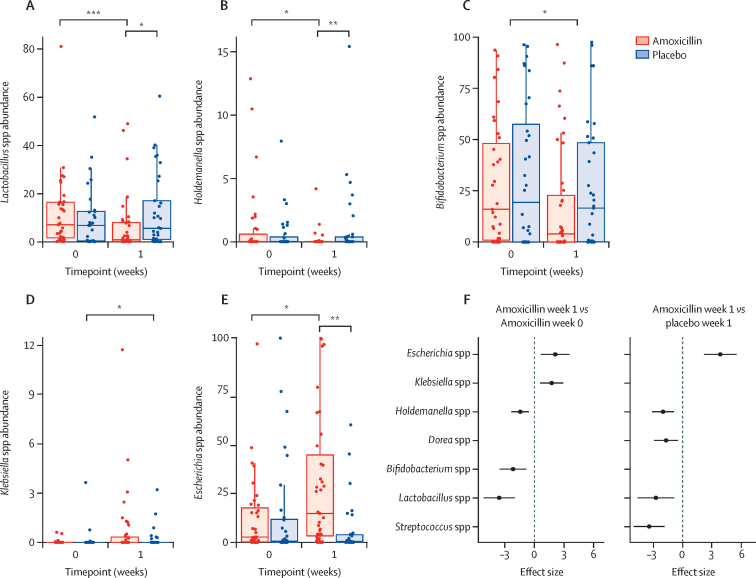
Effect of amoxicillin on the taxonomy of the gut microbiome from baseline (week 0) to week 1 (A–E) Significant taxonomic changes at the genus level with Benjamini-Hochberg-corrected q-values. Y-axes displays the relative abundance of that genus within the gut metagenome. (F) Coefficients (effect size) and 95% confidence intervals for significant taxonomic enrichment and depletion showing enrichment of *Escherichia* spp and *Klebsiella* spp at baseline and of *Escherichia* spp at week 1. All with q-values <0·05. Significant values and effect sizes are shown in appendix 2 (tab 3). *q<0·05. **q<0·01. ***q<0·001.

With this observation at the phylum level, we investigated which genera were depleted and enriched relative to baseline. We found that *Lactobacillus* spp (effect size –3·57, 95% CI –5·17 to –1·97, q=0·0003; figure 3A), *Holdemanella* spp (–1·43, –2·34 to –0·53, q=0·018; figure 3B), and *Bifidobacterium* spp (–2·16, –3·54 to –0·78, q=0·019; figure 3C) were the most depleted after 1 week of amoxicillin. *Klebsiella* spp (1·76, 0·56 to 2·96, q=0·029; figure 3D) and *Escherichia* spp (2·12, 0·64 to 3·59, q=0·034; figure 3E) were enriched over baseline samples. Two genera significantly increased and five genera decreased in the gut microbiomes of amoxicillin-treated children after 1 week relative to baseline and week 1 placebo group, or both (all p<0·01, q<0·05; figure 3F; appendix 2 tab 3). Therefore, amoxicillin treatment resulted in taxonomic changes immediately following treatment.

**Figure 4:**
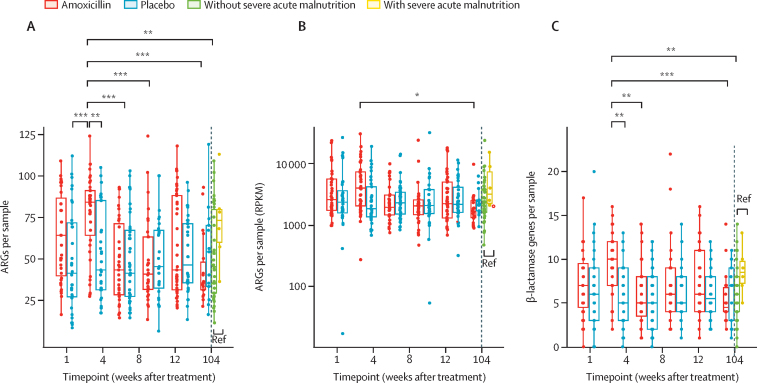

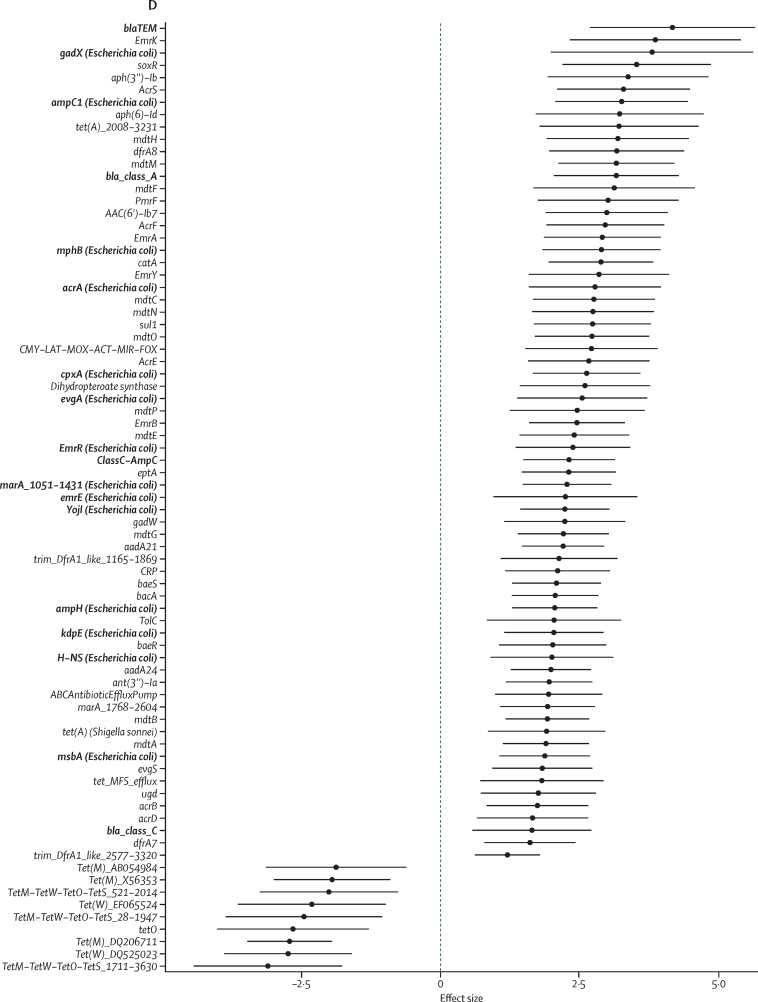
Effect of amoxicillin on ARGs in the gut microbiome (A) Number of ARGs per sample. (B) Total ARG RPKM. (C) Number of annotated β-lactamase genes per sample. (D) Enrichment and depletion at week 1. Significant values and effect sizes are shown in appendix 2 (tab 7). ARG=antimicrobial resistance gene. RPKM=reads per kilobase million. *p<0·05. **p<0·01. ***p<0·001.

It was previously shown that extended spectrum β-lactamase (ESBL)-producing Enterobacterales increased acutely after amoxicillin treatment.^21^ Given this finding and our observed increase in *Klebsiella* spp and *Escherichia* spp immediately following antibiotic treatment (figure 3D–F), we investigated whether the gut resistome also changed. At baseline, the amoxicillin group had 63·1 (95% CI 54·4–72·0) ARGs and the placebo group had 49·0 (39·8–58·0) ARGs (p=0·64). We found an immediate, significant increase in the number of ARGs (figure 4A) as well as their relative abundance in the gut metagenome after amoxicillin (figure 4B). The median number of ARGs in amoxicillin-treated children was 76·9 (95% CI 68·6–85·0) at week 1 compared with 52·0 (43·0–61·0) for placebo-treated children (estimated difference 24·9, 95% CI 12·4–37·4, p=0.0060; figure 4A; appendix 2 tab 4). This ARG increase was transient and by 3 weeks after amoxicillin completion was not significantly different from baseline (15·1, 2·5–27·7, p=0·48) and was similar between the two groups at week 4 (–0·7, –12·7 to 11·3, p=1·0). 2 years after treatment, ARG relative abundance was 2254 RPKM (95% CI 518·0–3989·0), which was significantly lower than the immediate post-amoxicillin increase (estimated difference 3790 RPKM, 95% CI 1748–5832, p=0·017; appendix 2 tab 5). We found that β-lactamase ARGs were specifically enriched within the gut resistome in amoxicillin-treated children over placebo-treated children (9·7 *vs* 5·9 genes, estimated difference 3·7, 95% CI 2·0–5·5, p=0.0014; figure 4C; appendix 2 tab 6). When controlling for age, MUAC, and WHZ, we found 68 ARGs significantly increased and nine ARGs significantly decreased in amoxicillin-treated children at week 1 relative to placebo-treated children at week 1 (figure 4D; appendix 2 tab 7). The enriched ARGs included 14 attributed to *Escherichia coli* and four predicted β-lactamases, consistent with the taxonomic increases we observed (figure 3E–F). However, we did not identify any ARGs significantly enriched in amoxicillin-treated children at 2 years. Thus, we found significant ARG enrichment 1 week after treatment, which resolved by 3 weeks after finishing treatment and did not persist at the end of the 2-year observation period.

**Figure 5:**
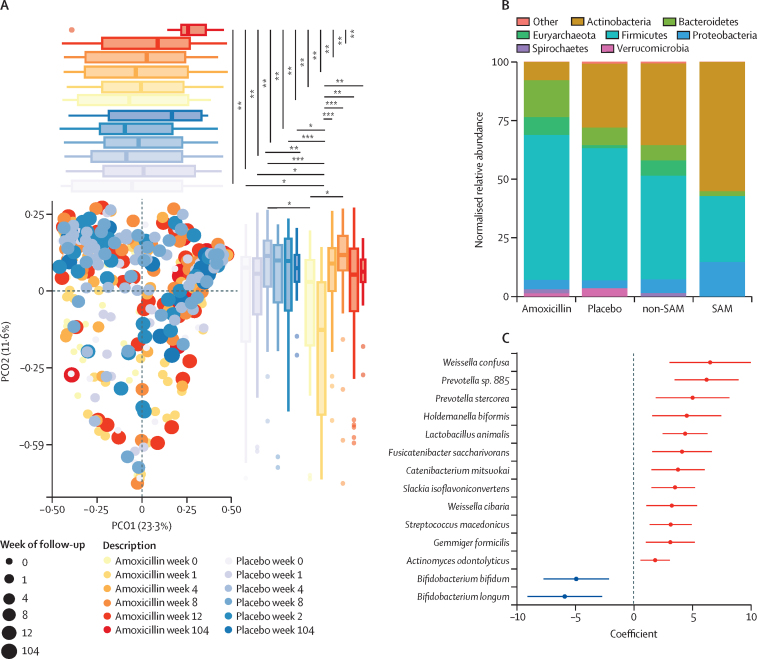
Effect of amoxicillin on long-term microbiome maturation (A) *p<0·05. **p<0·01. ***p<0·001. Bray-Curtis dissimilarity at the species level is plotted for the amoxicillin and placebo groups over time. Boxplots above and to the right of the figure demonstrate the distributions in PCO1 and PCO2, respectively. The number in parenthesis on the axes represents the variance explained with each PCO. (B) Taxonomic contribution at the phylum level is plotted for timepoint 104 samples. (C) Coefficients (effect size) and 95% CI for taxa significantly different between amoxicillin-treated and placebo-treated children. *q<0·05. **q<0·01. ***q<0·001. Relative abundance by species is shown in appendix 3 (p 7). Significant values and effect sizes are shown in appendix 2 (tab 8–10).

We hypothesised that 1 week of amoxicillin might have short and long-term effects on the gut microbiome relative to placebo-treated children because we observed taxonomic changes immediately thereafter (Figure 2, Figure 3). Amoxicillin-treated samples from week 1 differed from all other timepoints in principal coordinate 2 in Bray-Curtis dissimilarity (figure 5A, appendix 2 tab 8). Similarly, the gut microbiome of amoxicillin-treated children 2 years after treatment differed significantly from all others except placebo-treated children at week 104 in principal coordinate 1 (figure 5A). 2 years after treatment, we found that amoxicillin-treated children had increased gut microbiome richness over placebo-treated children (60·7, 95% CI 54·7–66·6) species versus 36·9 (29·4–44·3; estimated difference 23·8, 95% CI 14·1–33·4, p=0·0001; figure 2A).

We hypothesised that the older amoxicillin-treated and placebo-treated children (ie, the children treated at the 2-year timepoint) would have increased microbiome diversity over younger children with and without SAM. Consistent with our hypothesis, amoxicillin-treated children had increased microbiome richness (figure 2A) and diversity (figure 2B) over non-SAM children 2 years younger on average; however, the placebo-treated group did not in this non-randomised analysis (appendix 2 tab 2). We found that the gut microbiomes of amoxicillin-treated children clustered together in principal coordinate 1, segregated from non-SAM controls (p=0·0064) and children with SAM who were 2 years their junior (p=0·036; appendix 3 p 5). Although Bray-Curtis dissimilarity was not statistically different for amoxicillin-treated versus placebo-treated children at this timepoint (p=0·19; figure 5A), placebo-treated children did not significantly differ from children 2 years their junior suggesting incomplete microbiome maturation (p=0·42). These reference cohorts (SAM and non-SAM controls) did not significantly differ from children at the start of the original placebo-controlled trial based on Bray-Curtis dissimilarity (p=0·067; appendix 3 p 5). The gut microbiomes of amoxicillin-treated children at the 2-year timepoint had the lowest relative abundance of the phylum Actinobacteria: 8% *vs* 27% for placebo-treated children (p=0·36), 35% for non-SAM controls (p=0·024), and 55% for SAM controls (p=0·024; figure 5B; appendix 2 tab 9). Actinobacteria, including the members of the family Bifidobacteriaceae, are common during infancy (appendix 3 p 4) but rare once a child is aged 3 years and into adulthood.^8^ We identified 12 species enriched and two taxa depleted in amoxicillin-treated children relative to placebo at the 2-year timepoint (figure 5C; appendix 2 tab 10). *Weissella confusa* was the most enriched species differentiating amoxicillin-treated from placebo-treated children (effect size 6·6, 95% CI 3·0–10·1, q=0·0057; appendix 3 p 7). *Weissella* spp are Firmicutes, which is the most abundant phylum in amoxicillin-treated children (figure 5B) as well as one of the most abundant phyla in healthy children and adults.^8^, ^9^, ^22^ *Prevotella* spp 885 (6·24, 3·46–9·02), q=0·0003; appendix 3 p 7) and *Prevotella stercorea* (5·05, 1·85–8·25, q=0·026; appendix 3 p 7) were also significantly increased in the gut microbiomes of amoxicillin-treated children (appendix 2 tab 10). *Prevotella* spp and other members of the Bacteroidetes phylum increase in abundance at cessation of breastmilk consumption.^9^, ^22^ *Bifidobacterium longum* (–4·90, –7·76 to –2·05, q=0·012; appendix 3 p 7) and *Bifidobacterium bifidum* (–5·89, –9·14 to –2·63, q=0·0072; appendix 3 p 7) were greatly reduced in amoxicillin-treated children relative to placebo-treated children. Two (9%) of 22 amoxicillin-treated infants and six (46%) of 13 placebo-treated children had detectable *B longum* in the gut microbiome at 2 years (Fisher's exact test p=0·032; appendix 3 p 7). Only one (5%) of 22 amoxicillin-treated children had any *B bifidum* versus six (46%) of 13 placebo-treated children (p=0·0059; appendix 3 p 7). The relative abundance of three *Bifidobacterium* spp, including *B longum* and *B bifidum*, were increased in placebo-treated children relative to children without SAM who were 2 years their junior (appendix 3 p 7). Therefore, while amoxicillin-treated infants demonstrated microbiome maturation with accumulation of older child or adult-like taxa^8^, ^9^ and depletion of infant-specific taxa,^22^, ^23^ just under half (six [46%] of 13) placebo-treated children maintained *Bifidobacterium* spp at abundances higher than younger children without SAM.

## Discussion

We demonstrate a benefit of amoxicillin on maturation of the gut microbiome in children treated for uncomplicated SAM. The gut microbiome of amoxicillin-treated children was enriched for ARGs and potentially antibiotic resistant *E coli* and *Klebsiella* spp, including potentially ESBL-producing Enterobacteriaceae^21^ at the end of the 7-day treatment; however, this adverse effect resolved within 3 weeks. Although placebo-treated children also had anthropometric improvement from baseline over the 12-week and 2-year follow-up, their gut microbiome maintained an age-regressed conformation relative to amoxicillin-treated and younger children without SAM. Our findings provide valuable information about the potential risks and benefits of routine amoxicillin administration as a component of the care package for children with uncomplicated SAM.

The cost–benefit calculation for providing routine antibiotic treatment to children with uncomplicated SAM requires consideration of both the potential benefits of possible immediate individual weight gain, nutritional recovery, decreased hospitalisation, and lower mortality with the potential future risk of infections with antibiotic-resistant organisms and their spread within the community.^6^, ^24^ The microbiome and antibiotic resistome results presented here are consistent with previous findings that amoxicillin can promote positive clinical outcomes in severely malnourished children.^4^, ^25^ Additionally, amoxicillin could help the gut microbiota adapt to a more mature diet by reducing the abundance of taxa specialised for milk utilisation such as *Bifidobacterium* spp. Indeed, amoxicillin has been shown to reduce *Bifidobacterium* spp abundance^26^ and would be expected to enrich for resistant Gram-negative organisms exactly as we observed. Accordingly, amoxicillin treatment may act as a reset to allow anaerobes and solid-food utilising microbes to increase their abundance in the gut.

WHO recommends routine antibiotics in the treatment of uncomplicated SAM, although there has been much debate on the long-term consequences of such practice.^27^ The major argument against the use of routine antibiotics in vulnerable populations is the loss of microbial diversity and selection for resistant pathogens, which could be particularly severe in patients who are immunocompromised and in settings where health-care infrastructure is inadequate.^1^, ^6^ Immediately after amoxicillin treatment, we observed increases in overall ARGs and potentially antibiotic resistant *Klebsiella* spp and *Escherichia* spp. Fortunately, the negative consequences of microbiome and resistome perturbation appeared to be short-lived, having disappeared by 3 weeks from the conclusion of treatment, and we further observed several unexpected long-term benefits to amoxicillin treatment, including improved long-term microbiome richness, diversity, and maturation. In healthy children older than 2 years, some adult-like stability and resilience to antibiotics can be expected.^9^ Malnourished children, however, exhibit an age-regressed microbiome that is often composed of different taxa than those common to a healthy child or adult.^10^, ^11^ For example, the gut microbiome of children with SAM is enriched for Proteobacteria relative to children without SAM living in the same geographical area.^10^ Children with SAM also often have decreased abundance of Firmicutes relative to those without SAM.^10^ Broad spectrum antibiotics could further deplete commensal microbial defences and inhibit the immune system.^6^ The increase of potential pathogens in children with an age-regressed microbiome and immune health could have negative consequences leading to invasive antibiotic-resistant bacterial infections.^6^ Nutritional interventions such as ready-to-use therapeutic foods have been shown to temporarily improve the maturation of the gut microbiome and anthropometric scores; however, this improvement is not always sustained.^10^, ^11^ Over the past 5 years, rationally designed microbiota-directed therapeutic foods have shown promise in microbiome restoration and improvement in anthropometric scores of children with moderate and severe acute malnutrition.^28^, ^29^ Thus, modifying the gut microbiome could be a core component in optimising treatment of acute malnutrition.

Our study has limitations. First, our follow-up cohort was a small subset of participants in the parent trial and, although we observed robust differences in gut microbiome changes after amoxicillin treatment relative to placebo, these data would need to be validated in larger cohorts. Similarly, our conclusions about increased microbiome maturity relative to *Bifidobacterium* spp abundance are based on small sample sizes at the 2-year timepoint of only 13 placebo-treated children. Second, our study population included children from Niger; it is important to note that children from other locations with different baseline gut microbiomes might respond differently to antibiotic interventions. Third, because of the gap between the 12-week and 2-year timepoint wherein we did not collect data, we cannot say that the amoxicillin-treated and placebo-treated children had equivalent environmental challenges, antibiotic prescriptions, and access to nutrition. We additionally do not have this information for the non-SAM and SAM reference controls whose environment, age, and exposures might differ from the original trial, so we cannot conclude there are no additional confounders in that non-randomised analysis at 2 years. Finally, we have not directly compared antibiotic regimens (eg, amoxicillin, trimethoprim–sulfamethoxazole, or azithromycin), so future studies directly comparing these interventions on the gut microbiome and resistome are warranted.^30^

## Data sharing

Processed shotgun metagenomic sequencing files are shared on NCBI BioProject (https://www.ncbi.nlm.nih.gov/bioproject/) and can be accessed by searching: PRJNA739008. Custom code is available on GitHub at https://github.com/DJSchwartzLab/NigerSAM.

## Declaration of interests

We declare no competing interests.
