# Correction to *Lancet Microbe* 2023; 4: e931–42

**DOI:** 10.1016/S2666-5247(24)00096-X

**Published:** 2024-05

*Drew J Schwartz, Amy Langdon, Xiaoqing Sun, et al. Effect of amoxicillin on the gut microbiome of children with severe acute malnutrition in Madarounfa, Niger: a retrospective metagenomic analysis of a placebo-controlled trial.* The Lancet Microbe *2023;* ***4:*** *e931–42*—In figure 4 of this Article, the x-axis labels were incorrect and the 0 week timepoint label was missing in parts A*–*C. This correction has been made to the online version.

