# Supplementary material for: Effect of amoxicillin on the gut microbiome of children with severe acute malnutrition in Madarounfa, Niger: a retrospective metagenomic analysis of a placebo-controlled trial
Source: Lancet Microbe. 2023 Nov;4(11):e931–42. doi: 10.1016/S2666-5247(23)00213-6 (PMC10620469; doi:10.1016/S2666-5247(23)00213-6)
Supplement: Supplementary appendix 1 [file mmc1.pdf]

THE LANCET
Microbe

Supplementary appendix 1

This appendix formed part of the original submission and has been peer reviewed. We post it as supplied by the authors.

Supplement to: Schwartz DJ, Langdon A, Sun X, et al. Effect of amoxicillin on the gut microbiome of children with severe acute malnutrition in Madarounfa, Niger: a retrospective metagenomic analysis of a placebo-controlled trial. *Lancet Microbe* 2023; published online Oct 19. https://doi.org/10.1016/S2666-5247(23)00213-6.

APPENDIX

Supplementary Methods

Study design

The criteria for SAM was weight-for-height Z (WHZ) score of -3 or less or a mid-upper arm circumference (MUAC) less than 115mm or both (5, 13). The patients were given outpatient standard of care (14) as well as albendazole, vitamin A supplementation, and malaria and anemia treatment if indicated (5). All enrolled children were given 170 kcal/kg/day of Plumpy'nut® (Nutriset, France) and a seven-day regimen of either 80 mg/kg/day amoxicillin or placebo divided twice a day. Children were transferred to inpatient management with any weight loss greater than 5% or if they had no weight gain after 2 weeks or per study clinician discretion for clinical decompensation and lost to follow up.

Supplementary Results

MUAC, change in MUAC, WHZ, and change in WHZ score increased over the course of the 2-year study period for both groups (Fig. S1). We observed lower MUAC (Fig. S1A, n= 381 observations, estimate -0.26 (95% CI -0.49:-0.04), p=0.024) and smaller change in MUAC (Fig. S1B, n=314 observations, estimate -0.2 (95% CI -0.39:-0.01), p=0.037) for placebo-treated children versus amoxicillin-treated children. Placebo treatment did not significantly impact WHZ score (Fig. S1C, n=381 observations, estimate -0.15 (95% CI -0.44:0.14), p=0.32), but was associated with a significantly decreased change in WHZ compared to amoxicillin-treated children (Fig. S1D, n=314 observations, estimate -0.39 (95% CI -0.66:-0.11), p=0.0060, appendix 2 tab 11).

We hypothesized that amoxicillin might also have long-term impacts relative to other children in the area. We collected clinical and anthropometric metadata (appendix 1) and a single fecal sample from 38 children without SAM and 6 children with SAM matching the ages of the amoxicillin- and placebo-treated children at baseline to evaluate this hypothesis (Table). To determine age-discriminatory microbial taxa in children without SAM in Niger, we modeled the chronological age of these 38 children without SAM with their gut microbiome composition using MaAsLin2. Within that group, as age increased, the relative abundance of the family *Bifidobacterium* decreased dramatically in the gut metagenomes while anaerobes within the families *Clostridiaceae* and *Eubacteriaceae* increased (Fig. S2, appendix 2 tab 12). We then used Random Forest classification on these children without SAM to identity species-level changes to the gut microbiome accompanying increasing age (Fig. S3A). Consistent with predictions from MaAsLin2 (Fig. S2), we found the most microbiome features accompanying chronological age were members of these families (Fig. S3B, appendix 2 tab 13). Specifically, the most important features to predict chronological age from microbiome age were *Clostridium ventriculi*, *Eubacterium sp. CAG 251*, *Roseburia hominis*, *Bifidobacterium bifidum*, *Coprococcus eutactus*, and *Bifidobacterium longum*. This cross-sectional analysis reproduced what has been observed in other studies regarding the longitudinal development of the microbiome with *Bifidobacterium bifidum* and *Bifidobacterium longum* declining with age (9). However, because we do not have multiple fecal samples from these individuals nor do we have many samples from children over 30 months of age, we cannot confirm that they would

experience the same microbiome trajectory. Therefore, we identified local changes in the gut microbiome accompanying age in a cohort of local children without SAM.

Supplementary Figures

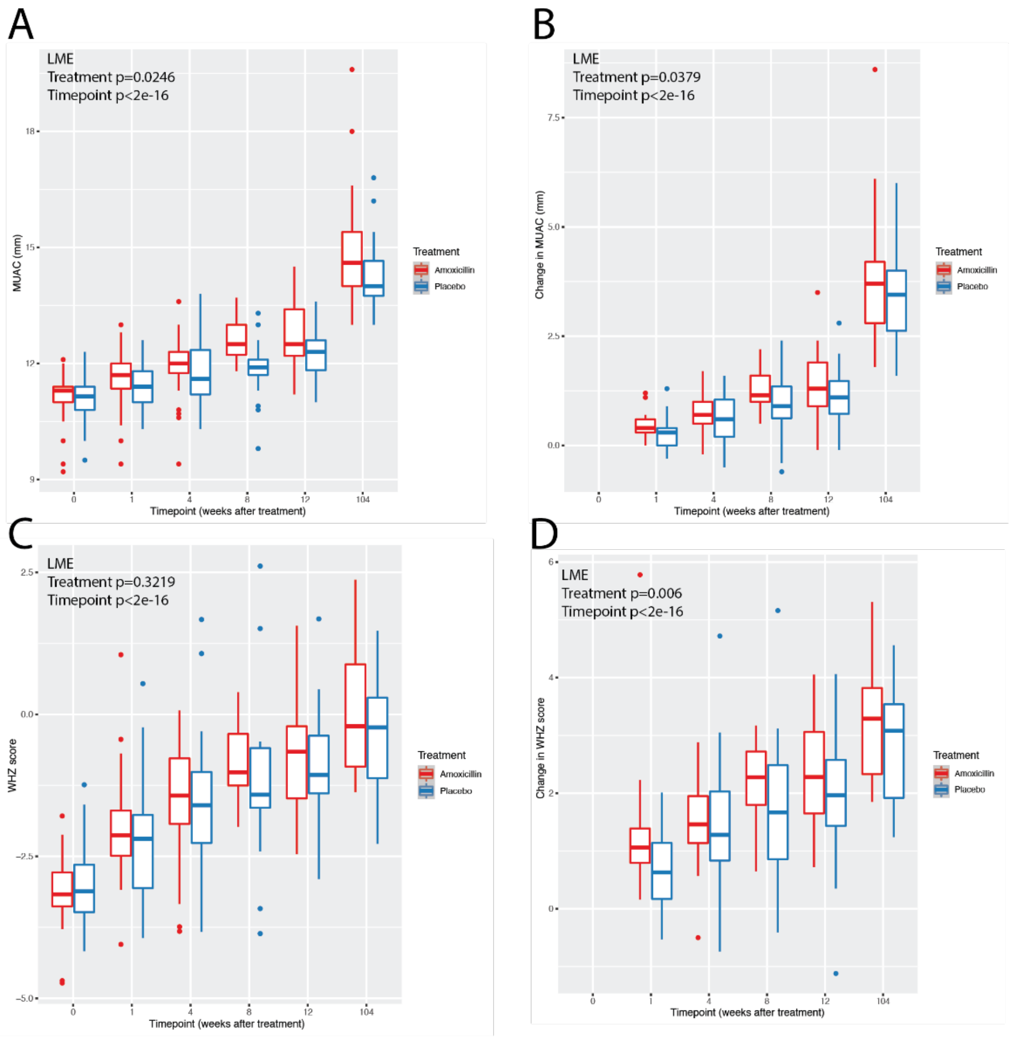

Supplementary Figure 1. Anthropometric measures improve over the two-year period. A) MUAC and change in MUAC from baseline (B) improve for both groups, but more so for amoxicillin treated children during the 2-year observation period. C) WHZ score improved for both groups, but no significant difference was observed between treatments. D) WHZ score improvement from baseline was higher for amoxicillin-treated vs. placebo-treated children. P values determined using linear mixed effect model with participant as random effect followed by ANOVA (timepoint and treatment p values displayed on each panel).

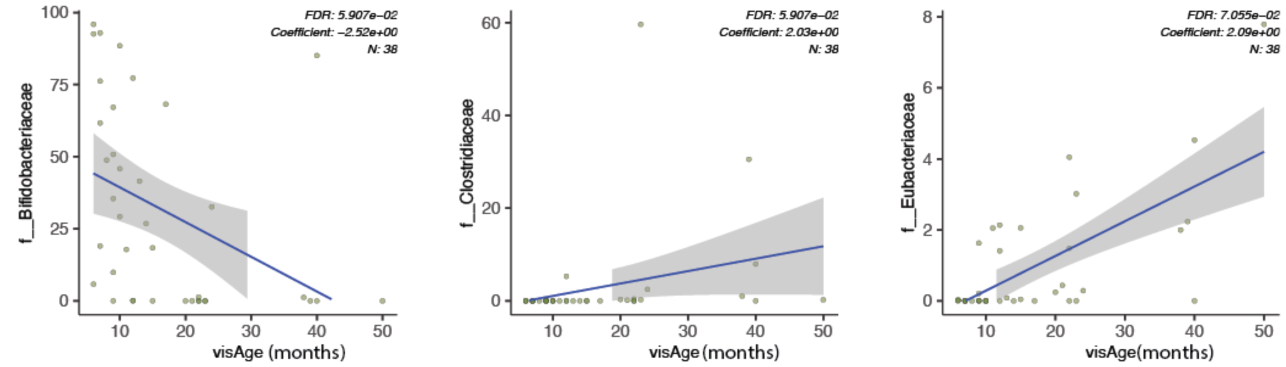

Supplementary Figure 2. Taxa significantly associated with age in non-SAM reference cohort. Species-level composition of children without SAM was determined using MaAsLin2. These 3 taxa are the only significant changes at the family level related to sampling age. FDR value corrected with BH is shown as well as the coefficient of the effect.

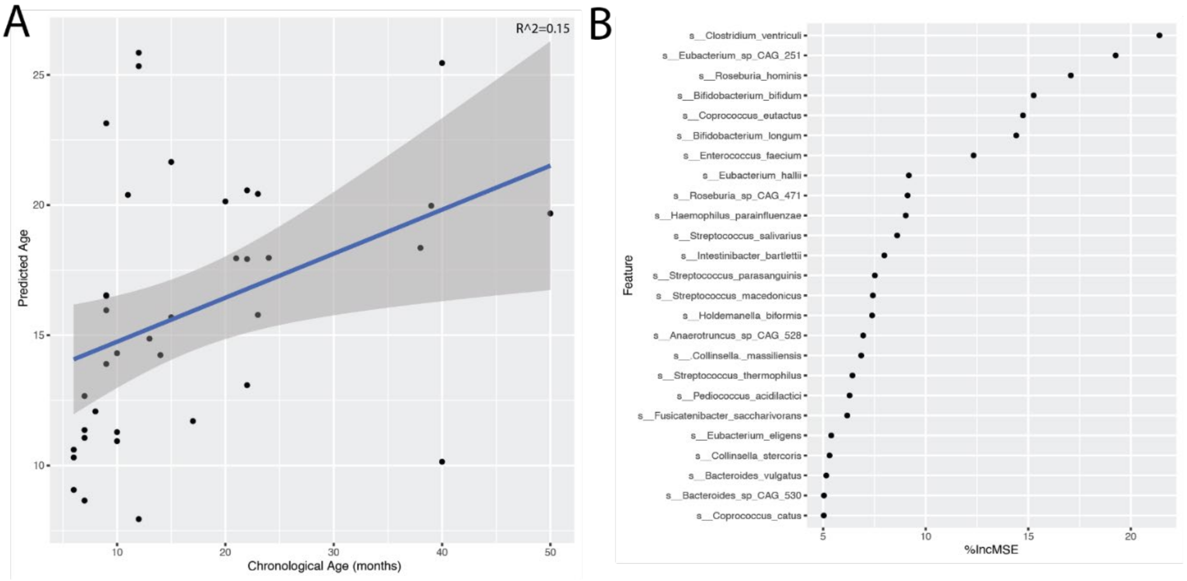

Supplementary Figure 3. Random Forest prediction of species contributing to microbiome maturation in Nigerien children without SAM. A) Predicted age versus chronological age was determined using gut microbiome species using Random Forest prediction. B) The most important features for reducing the mean square error (MSE) are displayed.

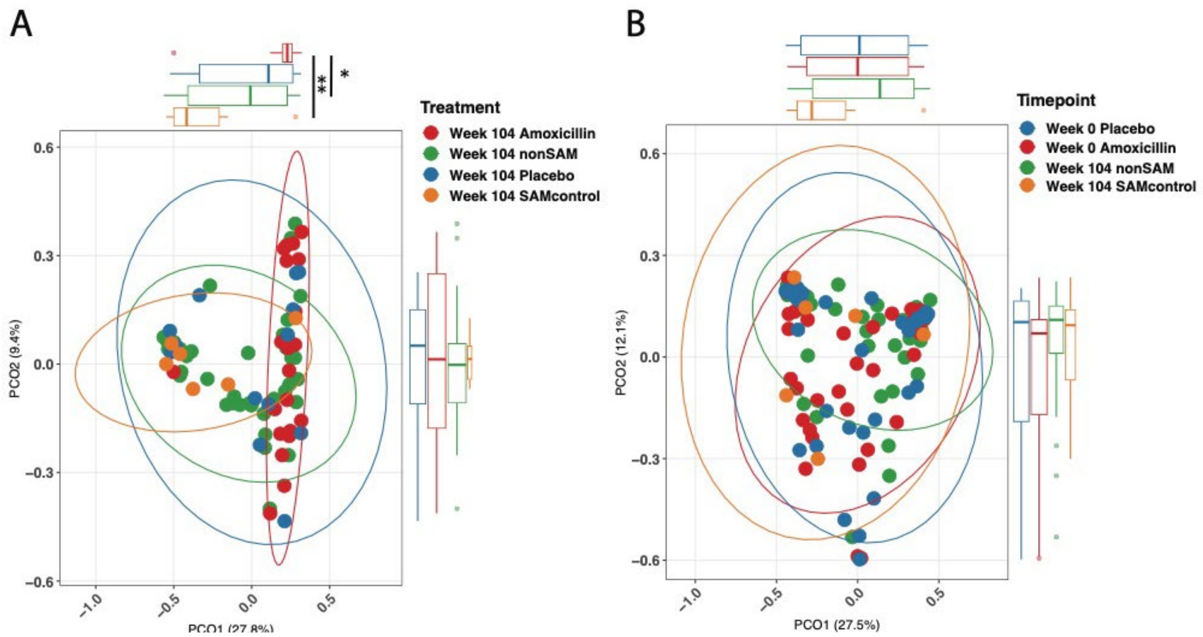

Supplementary Figure 4. Amoxicillin-treated children 2 years after treatment differ significantly from younger children, but placebo-treated children do not. A) Bray-Curtis dissimilarity at the species level of only samples at the 104 timepoint. B) Bray-Curtis dissimilarity performed on baseline samples from placebo- and amoxicillin groups compared to SAM and non-SAM reference controls. PCO differences determined overall by PERMANOVA and pairwise Wilcoxon test with BH correction for each PCO independently. *, p<0.05, **, p<0.01

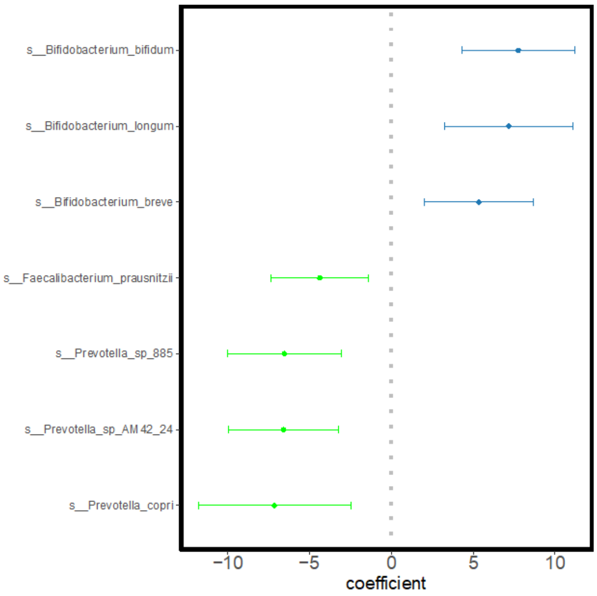

Supplementary Figure 5. Placebo-treated children have significantly increased abundance of *Bifidobacterium* spp. relative to children without SAM. Coefficient of significantly increased species between placebo-treated and non-SAM children are shown in blue while those decreased shown in green. Generalized linear mixed effect models with MaAsLin2 controlled for visit age and anthropometry. All with q<0.05, appendix 2 tab 15.
